# Data on chemical composition of soil and water in the semiarid wetland of Las Tablas de Damiel National Park (Spain) during a drought period

**DOI:** 10.1016/j.dib.2018.04.085

**Published:** 2018-09-12

**Authors:** Héctor Aguilera, Luis Moreno Merino

**Affiliations:** Geological Survey of Spain, Ríos Rosas 23, 28003 Madrid, Spain

## Abstract

Las Tablas de Daimiel National Park (TDNP) is a Ramsar Mediterranean wetland area designated as Biosphere Reserve by Unesco. The whole system dried out during a long drought period in the past decade (2006–2009) and a smouldering peat fire took place in 2009. The physical and chemical properties of sediments were significantly disturbed by the fire. To date, the ecological system has not fully recovered from the impact. We present a raw data collection of the chemical composition of soil, groundwater and surface water sampled over four consecutive years in 2006–2010. The data include major and minor anions and cations, nutrients and heavy metals. Sampling points were located inside and outside TDNP both upstream as well as downstream. The information provided can be used to analyse the medium and long term impact of drought and smouldering fire to the TDNP environment. It is also a baseline for hydro-ecological modelling of the impact of climate change in arid and semiarid wetlands to develop adaptive management strategies.

**Specifications table**

**Table:** 

Subject area	Chemistry
More specific subject area	Hydrochemistry in wetlands
Type of data	Tables, figures, spatial map (format.kmz)
How data was acquired	Field sampling, chemical analysis
Data format	Raw, analysed
Experimental factors	Standard preservation procedures until the analysis.
Experimental features	Major and minor hydrochemical compounds, pH, electrical conductivity, soil organic matter, soil total nitrogen, soil available phosphorous and soil carbonates were measured.
Data source location	Biosphere Reserve of “Parque Nacional de Las Tablas de Daimiel” and its surroundings
Data accessibility	Data is provided with this article

**Value of the data**

• The dataset could serve as a benchmark to assess the medium and long term impact of drought and smouldering peat fire on groundwater quality and dependent ecosystems in the TDNP area.
• The dataset provides baseline to study the functioning of the wetland-aquifer system and the properties of the soil for solute storage and transmission.
• The dataset could help explaining the biodiversity changes currently observed and distinguish effects due to the fire from those related to other factors such as pollution and climate change.
• In a global change context, the dataset supports understanding of human and climate driven degradation processes in arid and semiarid wetlands and how to develop adaptive management strategies.

## 1 Data

**Fig. 1:**
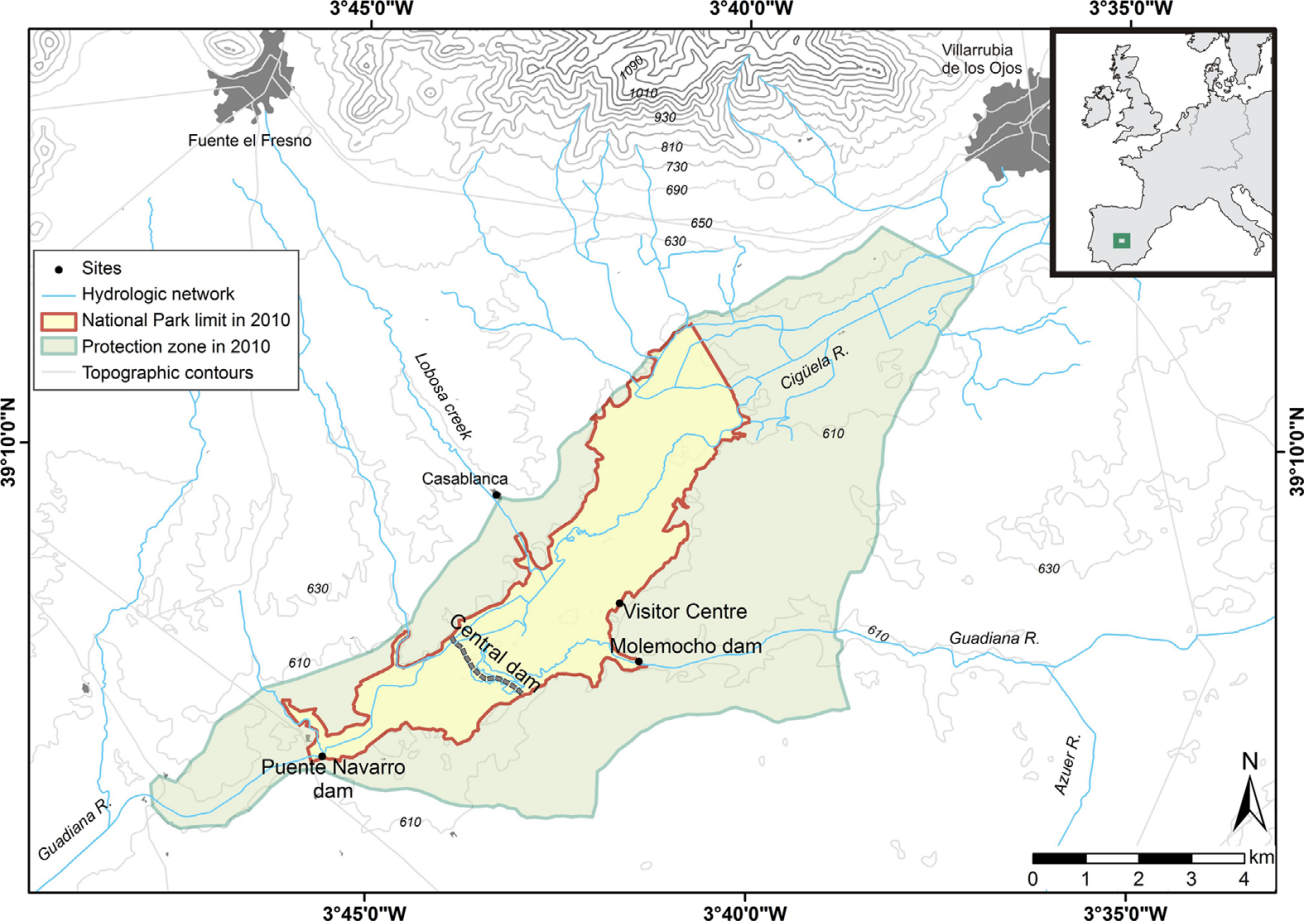
Geographical study site map of Las Tablas de Daimiel National Park (TDNP). The extent of TDNP and its Protection zone correspond to the limits in 2006–2010 when total TDNP surface was 1928 ha, two thirds of current 3030 ha.
3) Chemical composition of 1:5 soil-water extracts of wetland soils. Wetland desiccation allowed to sample dry sediments and quantify the amount of leachable solutes. These solutes could be washed by infiltrating water and transported to the aquifer.
4) Soil nutrient and organic matter content that condition the growth of invasive plants species and smouldering fire start and spread.

The dataset describes the chemical composition of soils, groundwater and surface water in TDNP over four consecutive years in 2006–2010 during which a severe drought made the wetland dry out leading to a smouldering peat fire in 2009. The dataset is divided into four groups according to the type of sample (Table 1):

1) Inorganic chemical composition of groundwater in the wetland area. Under natural conditions, groundwater supported wetland's hydroperiod throughout the year, but due to excessive pumping for irrigation the wetland turned into a recharge area.
2) Inorganic chemical composition of surface water. Main input sources are the Cigüela River, affected by treated wastewater spills from the village of Villarrubia de los Ojos (Fig. 1), and artificial flooding with groundwater pumped inside the Park.

**Table 1:** Summary of the type and volume of data provided.

	Groundwater	Surface water	Soil-water extracts	Solid soil matrix
Number of points	17	8	40	40
Number of samples	244	93	143	140
Number of parameters	28	28	17	4
Sampling periods	2003–2004; 2006–2010	2003–2004; 2006–2010	July 2006;	July 2006;
			February and April 2009	February and April 2009

Interpretations of data analyses have been published in different articles [1], [2], [3], [4]. Overall, the results show that TDNP is an eutrophic carbonated environment with high salinity and large topsoil nutrient accumulation controlled by reed dynamics [2]. Groundwater salinization and increased nutrient content are the hydrochemical footprints of degradation [3]. Organic carbon content is identified as one of the main controlling factors for smouldering peat fires [1].

## 2 Experimental design, materials, and methods

### 2.1 Study area description

TDNP is located in semi-arid central Spain in the confluence of the Cigüela and Guadiana Rivers (39°09′N, 3°40′W; Fig. 1). The park covers an area of 3030 ha of which approximately 1800 ha are subjected to flooding. The former natural wetland of TDNP was originated at the outflow boundary of the 15.000 km^2^ Upper Guadiana Basin and the 5.500 km^2^ Mancha Occidental groundwater system.

### 2.2 Sample collection and analytical procedures

**Fig. 2:**
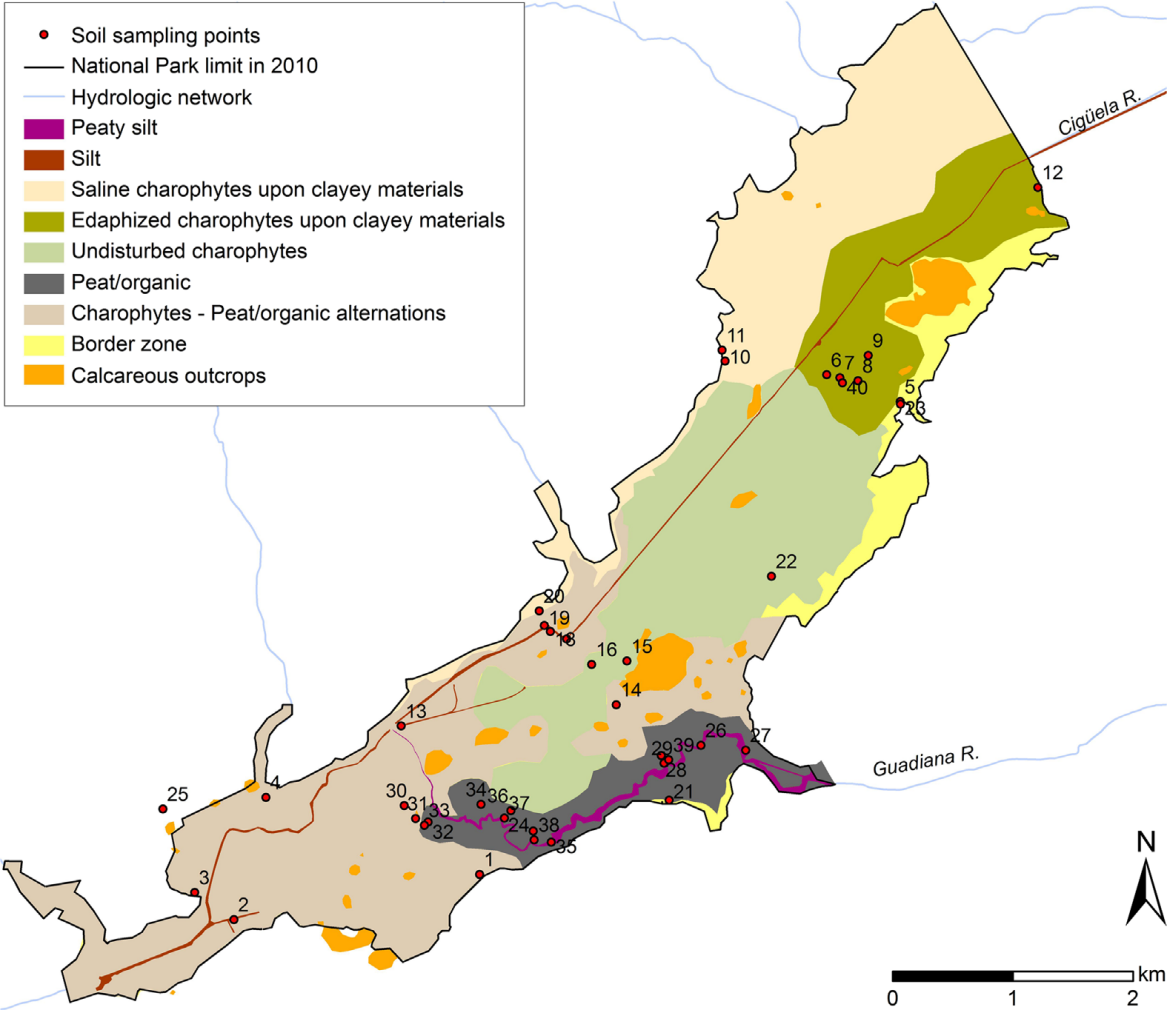
Location of sampling points for soil chemical analysis and spatial distribution of dominant soil types as described in [4].

The main soil sampling campaign was carried out between July 25th and 27th in 2006. Disturbed soil samples were taken every 20 cm, from the topsoil to a maximum of 120 cm deep, in 25 points located along four transects, three transversal and one longitudinal (Fig. 2). This experimental design was conditioned by accessibility but the arrangement of sampling points accounted for the variability associated to main water flux directions (NE-SW and E-W) as well as soil types and microtopographic variations inside the system. Topsoil effloresces were sampled at three points and only analysed for leachable solutes. Additionally, 15 points were sampled in February and April 2009 for specific peat chemical analysis (points numbered from 26 to 40 in Fig. 2).

Samples were collected with an Eijkelkamp P1.01 auger and kept in plastic bags. They were air dried before their analytical processing. Soil chemical analysis was performed according to standard ISRIC (International Soil Reference and Information Centre) methods [5]. Soil solutes were determined in 1:5 soil-water extracts: sodium and potassium (atomic emission spectrophotometry), calcium, magnesium, sulphate, chloride, bicarbonate, nitrate, nitrite, ammonium, phosphate and silica (absorption spectrophotometry), carbonate (volumetric analysis) and boron (inductively coupled plasma atomic emission spectrometry [ICP-AES]). The electrical conductivity of the 1:5 soil-water extracts was measured by electrometric analysis. In addition, the following complementary analyses were determined on the solid matrix: pH in 1:2.5 soil suspensions both in water and 1 M KCl solution (potentiometric determination), organic carbon (Walkley-Black method), nitrogen (Kjeldahl method), available phosphorus (Olsen method) and carbonates (Piper acid neutralization). Organic carbon is expressed as organic matter multiplying by the Von Bemmelen factor 1.724, which assumes that on average 58% of the organic matter content is organic carbon [6].

Analytical determinations on the solid matrix were performed at the laboratories of the Department of Soil Science of the Complutense University of Madrid, whereas soil-water extracts were analysed at the laboratory of hydrochemistry of the Geological Survey of Spain (IGME).

**Fig. 3:**
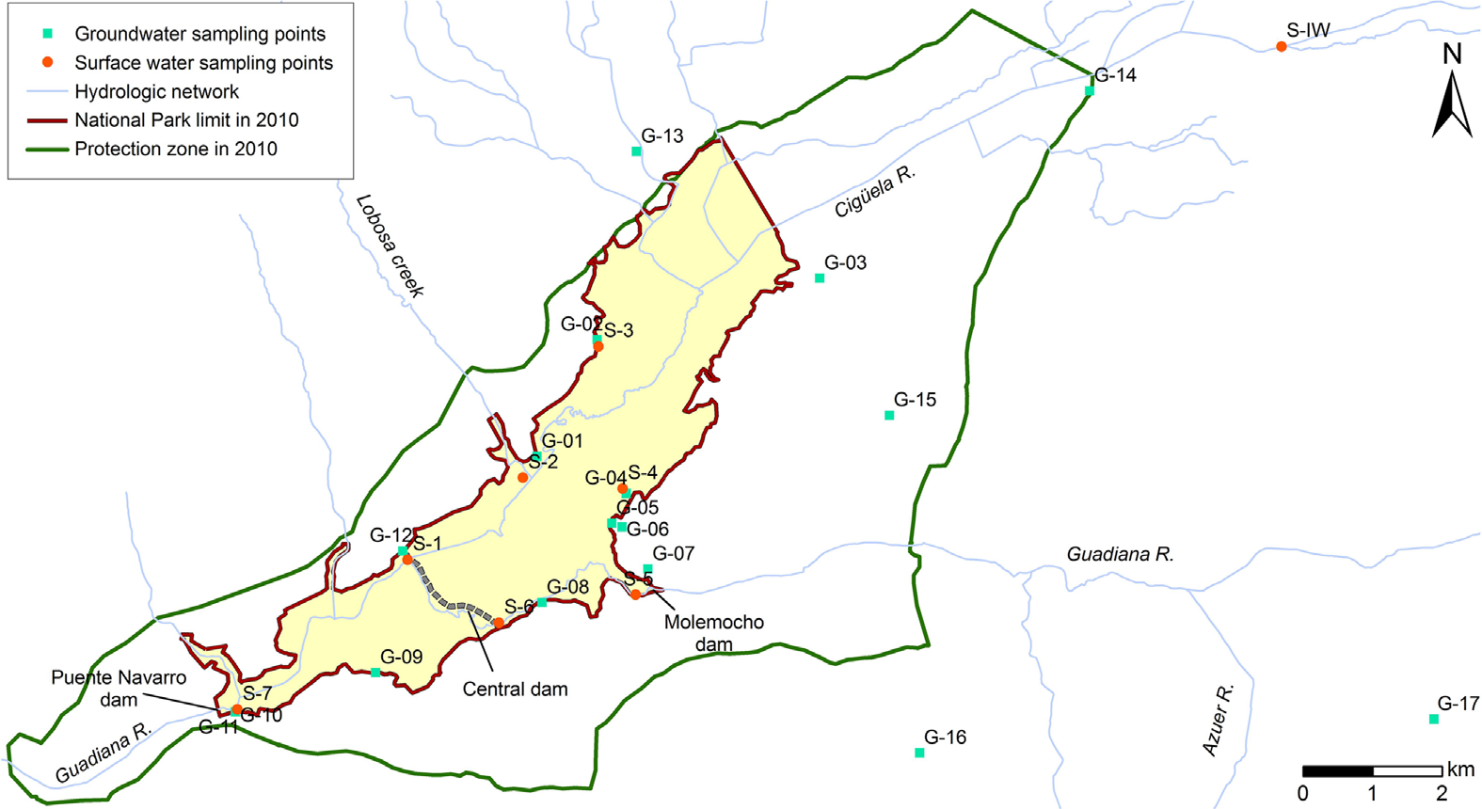
Location of sampling points for groundwater and surface water analysis.

The water sampling network consisted of 17 groundwater monitoring points (G) and 7 points for surface water (S) quality monitoring, located along the boundary of TDNP (Fig. 3, Table 1). Point S-IW, located at a gauging station in the Cigüela River upstream from TDNP, was used as a proxy for incoming surface water. All surface water sampling points and seven groundwater monitoring points were sampled on a monthly basis from April 2006 to October 2008. Surface water and groundwater samples from these points (except for S-5 and G-12) taken by IGME in July 2003 and June 2004 are also included. Point G-13 was dry most of the time throughout the studied period and only five samples could be collected. The remaining groundwater points were monitored at least twice a year, except for G-07, which was only sampled in September 2008 after construction. Further details on the characteristics of groundwater sampling points can be found in [3].

Polyethylene bottles were used to collect the samples, filled to the brim, preserved at 4 °C and delivered to the laboratory on the same day. No chemical preservatives were added and samples were not filtered. Electrometric analysis was used to determine the pH and the electrical conductivity of the samples in the field and in the laboratory. Major and minor anions and cations (atomic emission spectrophotometry for sodium and potassium, ICP-AES for boron, ionic chromatography for bromide, and absorption spectrophotometry with continuous flow autoanalyzer for other elements), oxidizability to potassium permanganate, heavy metals (atomic absorption spectrophotometry), and total organic nitrogen (Kjeldahl method) and carbon (UNE-EN 1484 method) were determined at the laboratory of hydrochemistry of the IGME.

## Appendix B Supplementary materials

The following KMZ files contain the Google maps of the most important areas described in this article.

Map KMZ file containing the Google map.
