# Supplementary material for: Data on chemical composition of soil and water in the semiarid wetland of Las Tablas de Damiel National Park (Spain) during a drought period
Source: Data Brief. 2018 Sep 12;19:2481–6. doi: 10.1016/j.dib.2018.04.085 (PMC6139365; doi:10.1016/j.dib.2018.04.085)
Supplement: Supplementary file 1 — Supplementary material [file mmc1.pdf]

CONFLICT OF INTEREST STATEMENT

Title: ***Soil and water chemical characteristics in the semiarid wetland of Las Tablas de Damiel National Park (Spain) during a drought period***

We, the authors of this data article, state that we don't have any conflict of interest with any researcher, editorial member, reviewer, organization, etc.

Sincerely,

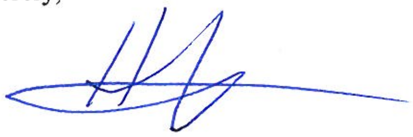

Héctor Aguilera (corresponding author)
Email: h.aguilera@igme.es
